# Assessing public perception of a sand fly biting study on the pathway to a controlled human infection model for cutaneous leishmaniasis

**DOI:** 10.1186/s40900-021-00277-y

**Published:** 2021-05-30

**Authors:** Vivak Parkash, Georgina Jones, Nina Martin, Morgan Steigmann, Elizabeth Greensted, Paul Kaye, Alison M. Layton, Charles J. Lacey

**Affiliations:** 1 York Biomedical Research Institute, Hull York Medical School, University of York, York, UK; 2 Department of Infection and Tropical Medicine, Sheffield Teaching Hospitals NHS Foundation Trust, Sheffield, UK; 3 School of Social Sciences, Leeds Beckett University, Leeds, UK; 4 Public involvement group participant, York, UK

**Keywords:** Controlled human infection model (CHIM), human challenge, Cutaneous leishmaniasis, *Leishmania*, Clinical study, Public involvement, PI, Expectations, Qualitative research

## Abstract

**Background:**

A controlled human infection model (CHIM) involves deliberate exposure of volunteers to pathogens to assess their response to new therapies at an early stage of development. We show here how we used public involvement to help shape the design of a CHIM to support future testing of candidate vaccines for the neglected tropical disease cutaneous leishmaniasis, a disease transmitted by the bite of infected sand flies in tropical regions.

**Methods:**

We undertook a public involvement (PI) consultation exercise to inform development of a study to test the safety and effectiveness of a sand fly biting protocol using uninfected sand flies (FLYBITE: ClinicalTrials.gov ID NCT03999970) and a CHIM using *Leishmania major*-infected sand flies (LEISH_Challenge: ClinicalTrials.gov ID NCT04512742), both taking place in York, UK. We involved 10 members of the public including a patient research ambassador and a previous CHIM volunteer. The session took place at The University of York, UK and examined draft study volunteer-facing material and included the CHIM study design, potential adverse events and therapeutic interventions at study endpoints. A discussion of the scientific, ethical, humanitarian and economic basis for the project was presented to the participants to provoke discourse. An inductive, thematic analysis was used to identify the participants’ key concerns.

**Results:**

Themes were identified relating to i) quality of volunteer-facing written information, ii) improving study design, and iii) factors to motivate involvement in the research. Group participants responded positively to the overall study aims. Initial concerns were expressed about potential risks of study involvement, but further explanation of the science and mitigations of risk secured participant support. Participants provided advice and identified improved terminology to inform the volunteer-facing material. Lastly, treatment options were discussed, and excision of any cutaneous lesion was favoured over alternatives as a treatment.

**Conclusion:**

The consultation exercise provided invaluable information which led to improved study design and enhanced clarity in the volunteer-facing material. The session also reinforced the need to maintain public trust in scientific rigour prior to initiation of any study. The investigators hope that this description strengthens understanding of PI in clinical research, and encourages its use within other studies.

**Supplementary Information:**

The online version contains supplementary material available at 10.1186/s40900-021-00277-y.

## Plain English summary

Our research team is designing a type of research study known as a controlled human infection model (CHIM). In CHIM studies, volunteers are exposed to infections on purpose and then studied to help understand diseases. Similar experiments, where humans are infected deliberately, have been used for hundreds of years to help test treatments. CHIM studies have already been used more recently to help test vaccines for diseases such as malaria.

The disease leishmaniasis, a disease affecting millions each year, is spread by the bite of an infected sand fly in tropical countries. There are currently no vaccines for leishmaniasis that are available for use in humans. It is thought that by using CHIM studies, new vaccines might be tested and then approved more quickly. Scientific researchers have had many discussions about how useful CHIM studies are, especially in terms of the science behind them, the safety of volunteers and the ethics of these studies. Researchers also understand how important it is to involve the public in designing and carrying out research, especially studies involving humans, to get an independent point-of-view. We have therefore involved the public, in some parts of designing this research, in a group discussion. We also included a person who has already taken part in a different CHIM study. These discussions have had an important effect and have changed how we plan to carry out our future research studies. We also hope that this description will encourage other researchers to include the public when planning future research.

**Supplementary Information:**

The online version contains supplementary material available at 10.1186/s40900-021-00277-y.

## Key lessons

- Controlled human infection models are not well known to the public, and it is important to educate the public in general as potential participants in research.
- Clarity of written and verbal information for volunteers is imperative in clinical research.
- The public representatives preferred excision biopsy over other treatment modalities. This stimulated discussion with clinical specialists and a consensus opinion was formed for this approach. This is a significant change to the project methodology influenced by the public involvement process. 
- Research conducted in the UK may not always accurately reflect stakeholders in other countries.
- There is a longstanding issue with diversity amongst research participants that is difficult to address.
- It is important to engage with technology (including social media) to both attract potential volunteers, but also to ensure engagement is maintained once a study commences.
- Public involvement is imperative to good clinical research as participants may add more holistic viewpoints that are not limited by the inherent bias of researchers.

## Background

The recognition of the concept of patient-centred care has proliferated since the mid-twentieth century [1]. The model for a patient-centred approach then progressed over the years, and has now become embedded within healthcare policy in the UK. With this focus on patient-centred healthcare, the discussion on stakeholder involvement in care has also moved to include research and clinical studies, with research being deemed integral to good healthcare. The consensus is therefore that high-quality research is dependent on public involvement [2].

Public involvement (PI) and engagement has been supported in recent years by both allocation of research funding specifically for PI activities and also by the creation of advisory bodies such as INVOLVE, championing public involvement initiatives and supported by the National Institute for Health Research (NIHR). The UK National Health Service (NHS) Health Research Authority has also published ‘The UK Policy Framework for Health and Social Care’ in conjunction with INVOLVE, which addresses public involvement in research including its remit in ethical review of studies [3, 4]. In addition, it has been demonstrated that public involvement at an early stage of study development benefits study design, increases recruitment and helps deliver high-quality research [5]. The benefits of PI, in introducing a more inclusive and collaborative approach to increase the quality of research, are well described [5]. This can result in more relevant and pragmatic research, that bridges the gap between researchers and the public, and can result in novel aspects to the research focus. This can occur through stimulating new discussions and by adding a unique viewpoint which can raise issues not previously anticipated by researchers [6, 7]. Extrapolating from this, there is some evidence to suggest PI may improve patient-facing materials as well as research literacy [7, 8]. As such, accountability for research studies can be strengthened, with the potential to involve research volunteers in this process [9]. It has also been postulated that research studies can become more cost-effective in the long-term as a result of PI [10].

We therefore describe here how our proposed research into the development of a controlled human infection model (CHIM), for the infectious disease leishmaniasis, was shaped by public involvement. This took the form of a PI consultation group session conducted by the research team at The University of York, with participants recruited from the UK. The terminology around stakeholder involvement in research is inconsistent, however we have adopted the use of the phrase ‘public involvement’ given its adoption by NIHR as evidence by the UK Standards for Public Involvement, following consultation in 2017 [4]. Furthermore, our PI group consisted of non-patient participants, and therefore the phrase ‘patient-public’ does not accurately reflect the background of our participant group.

A solution being adopted for many diseases, which our research team plan to adopt for leishmaniasis, is to develop a ‘human challenge model’ also called a ‘controlled human infection model’ (CHIM). This approach involves deliberate exposure of individuals to an infection, in a controlled setting, to better understand the disease and to test potential vaccines and treatments. There has been much ethical discussion of CHIM studies given the nature of deliberate infections [11]. The Hippocratic Oath has been quoted as a barrier to such studies, particularly with regard to a chequered history of deliberate infection in research in the early part of the twentieth century. During their refinement over the last 20 years, CHIM studies have contributed vital scientific knowledge that has led to advances in the development of drugs and vaccines [11, 12].

Leishmaniasis is listed by the World Health Organisation as a major neglected disease of poverty, affecting populations in low and middle-income countries (LMICs). It affects around 150 million people in 98 countries worldwide and is transmitted by a sand fly vector. To date there have been no autochthonous cases of leishmaniasis in the UK, although there have been a number of cases reported in some European regions [13]. It has been reported however in return travellers to the UK [14]. There are many species of *Leishmania* widely dispersed geographically and different species may cause disease that is manifest in the skin (tegumentary leishmaniasis) or the internal organs of the body (visceral leishmaniasis). The clinical spectrum of tegumentary leishmaniasis ranges from localised, usually self-healing, sores that result in a scar, (localised cutaneous leishmaniasis; LCL), to more widespread diffuse or disseminated disease. LCL scars can cause stigma, and disproportionately affect children. There are several drug treatments available to treat leishmaniasis, however various factors have limited their effectiveness including drug resistance, difficulty of use in LMICs, significant side effect profile and limited effectiveness of vector control programmes. New treatments and vaccines are urgently needed, but to date no vaccines have been approved. This development process would be immensely strengthened if there was a rapid, standardised method for the early evaluation of the efficacy of candidate vaccines. Deliberate exposure of humans to infectious pathogens has been studied as a method for better understanding disease and infection for several hundred years. More recently a similar approach has been used to investigate diseases where a deliberate infection would be beneficial in either better understanding a disease or to test potential therapies [15], including diseases as diverse as influenza [16], norovirus [17], malaria [18] and dengue [19]. Here, we describe how public involvement has helped shape the development of a CHIM for sand fly transmitted leishmaniasis. It is well known that sand fly transmission potentiates infectivity of *Leishmania* [20]. In addition, in experiments with *Leishmania* vaccines, it has been observed that these vaccines may be protective in mice that have been infected with *Leishmania* via needle, but may fail to protect mice infected with *Leishmania* via the natural sand fly vector [21]. As such a model resembling the natural infection in the wild, is needed to robustly test vaccines. Hence, our public involvement discussions covered issues related to the use of sand fly bite as a means of initiating leishmaniasis as well as leishmaniasis per se.

## Methods

### Project overview

**Fig. 1:**
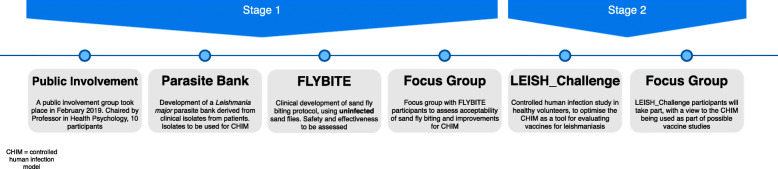
Schematic overview of incorporation of public involvement activities in the LEISH_Challenge project

To develop a CHIM for cutaneous leishmaniasis, a series of enabling studies (Stage 1) are required prior to the conduct of human infection studies (Fig. 1):

(i) a public involvement (PI) consultation group exercise which we describe here in detail,
(ii) development of a suitable challenge agent manufactured at Good Manufacturing Practices (GMP), described in detail elsewhere [22],
(iii) development of a protocol for sand fly bites on humans using pathogen-free sand flies (FLYBITE; ClinicalTrials.gov ID NCT03999970),
(iv) a focus group discussion at the end of the FLYBITE study.

Stage 2 of the project is a controlled human infection study for cutaneous leishmaniasis in healthy volunteers, (LEISH_Challenge; ClinicalTrials.gov ID NCT04512742), followed by a further focus group.

### Recruitment and PI consultation group location

Ethical approval for the PI consultation group conduct and all material (PI participant consent form, plain English summary, protocol, agenda, draft FLYBITE and LEISH_Challenge volunteer advertisement/volunteer information leaflets) was successfully obtained from the Department of Biology Ethics Committee, University of York (Ethics reference PK201812). Ethical approval from the NHS Research Ethics Committee was not deemed necessary as outlined by the NHS Health Research Authority decision tool [23] and according to INVOLVE guidance [24]. Therefore prior to the FLYBITE study, a PI consultation group with participants recruited widely, was carried out to make clinical investigators aware of the public perception of human challenge models as well as to inform the study design. The PI consultation group activity has been reported in this manuscript in accordance with the Guidance for Reporting Involvement of Patients and the Public (GRIPP2) reporting checklist [25] [see Additional file 2].

Participants were recruited to the PI consultation group through local and national advertisements including via patient advocate groups and University mailing lists. Participants interested in taking part in the PI consultation group were invited to make contact with the project team. Then, after an initial discussion, those participants who were interested were invited to attend. A total of 10 participants were recruited, 7 female and 3 male. They included student and staff volunteers from the University of York from a range of Science and Arts backgrounds, a previous CHIM volunteer from a UK centre, a previous vaccine study volunteer from a UK centre, a patient research ambassador, and lay volunteers from the local community. 6 participants were university educated, with 5 of these participants having undertaken further education in science fields. 5 participants were in current employment, and 4 participants were full-time students. Although age and ethnicity were not specifically recorded, the research team did note a lack of ethnic diversity amongst participants. All participants received remuneration to the value of £75 following their attendance, in addition to travel expenses. The remuneration was based on INVOLVE and NIHR guidance which was developed in conjunction with the UK Department of Health [26, 27]. Participants were fully consented for their involvement including for audio recording of proceedings and use of content in academic outputs. The consultation exercise took place over the course of 3 h, with all 10 participants present, and prior to ethical review of the interventional human studies. No patients previously treated for leishmaniasis were available for recruitment to the PI consultation group.

The PI consultation group took place at the University of York in February 2019 and was chaired by GJ, acting as an independent facilitator. Clinical team members were present for an initial summary of the project, including the chief investigator (CJL) and principal investigators (VP, AML). The clinical team then left the room to allow discussion between participants and the independent facilitator. Sample materials for the proposed project were provided in advance in order for participants to digest the information and judge the studies independently. In total, four documents were provided: a volunteer information leaflet for an uninfected sand fly biting study (FLYBITE), a volunteer information leaflet for a *Leishmania* CHIM (LEISH_Challenge), a plain English summary of the overall project and an advertisement poster for recruitment of volunteers.

Key roles of PI consultation group participants outlined in communication prior to attendance were to: i) consider the draft study protocols to identify any issues; ii) ensure that the designs of the clinical studies are optimal; iii) consider the ethical issues relating to the study; iv) consider the plain English summary of the project; and v) consider recruitment to the studies, and the feasibility of the inclusion criteria.

The content of the consultation exercise was guided by an initial presentation given by the clinical team on the overall aims of the CHIM study and the specific features of the disease, its treatment and current status within world health.

### Approach to data collection methodology

To structure the discussion, the initial plan was for a simple presentation to be given by the clinical investigators and then for the discussion to be held entirely between the independent facilitator and the PI consultation group participants, with the clinical investigators to be present to clarify any points arising. However, given the complex and unique nature of the LEISH_Challenge study, the prior research participation experience of some participants and the culture of openness and flexibility encouraged of participants, it transpired that participants wished to ask pertinent questions at each stage of the presentation. As such the format evolved to one where a particular aspect was presented, during which participants were able to interact with, and pose questions to, the clinical investigators. The independent facilitator then expanded the discussion at opportunistic time points both during and after each topic presentation as appropriate. Subsequent to this, the clinical team members left the room to allow open discourse between the participants and the independent facilitator. The agenda was not strictly adhered to in order to facilitate free-flowing discourse. Any topics not fully covered in the course of the consultation exercise were then addressed at the end of the session. Participants were also asked to voluntarily provide feedback at a later date, in an anonymous fashion if required, although this was not universally carried out.

### Analysis

The PI group session audio recording was fully transcribed verbatim by a colleague external to the study team. A qualitative thematic approach to data analysis was employed, using the methodology and processes advocated by Braun and Clarke [28], and assisted via the use of NVivo Pro software version 12 (QSR International Pty Ltd). The use of qualitative analysis software can, if used appropriately, facilitate data management, analysis and analytic rigor [29].

The research team present at the session included clinical team members (VP, CJ, AL), project manager (EG), and GJ who was the PI consultation group independent facilitator. CJ and AL have many years’ of experience with large clinical research studies, including vaccine studies and studies involving leishmaniasis. They both have some prior experience of PI within the development of these studies. VP is an early career researcher working on the project as a clinical research fellow, with a background as an infectious disease clinician and experience in treating cases of leishmaniasis in return travellers to the UK. GJ has several years’ experience as a health psychologist including significant experience with running PI groups and using PI in clinical research. PK is the senior scientific research lead for the project, and conceived the overall project, as well as having undertaken significant leishmaniasis research for many years. NM has several years’ experience as a qualitative health researcher and undertook transcription and coding of the transcript. MS is a participant from the PI consultation group who contributed to critical review of the manuscript and advised on the participant experience. PK and NM were not present during the consultation group.

Several team members were involved in the data analysis, independently listening to the audio-recording and reading the transcript several times (VP, GJ, NM), before coding, at a semantic level, was undertaken to identify and label the interesting items in the data (NM). A second member of the team independently checked a proportion of coding against the transcript (GJ). Together, and in an iterative process, they actively searched for and discussed where codes clustered together to develop the themes. In a further review of these themes, key themes and subthemes were generated and discussed with VP and the wider study team.

Thematic analysis is a highly flexible approach that can be used for engaging with, identifying and analysing the meanings inherent within qualitative data. The aim of this public involvement group was very much embedded within applied health research, with a straight-forward goal of summarising and interpreting the PI participants’ feedback and comments concerning the draft study volunteer-facing materials (including issues around study design, recruitment, feasibility, inclusion criteria, ethical issues, and the plain English summary). Therefore, an inductive (data-driven) approach to coding and analysis was undertaken to identify and prioritise the participants’ key concerns and generate the themes following, in a non-linear process, the six key stages that Braun and Clarke recommend [28].

## Results

All 10 participants were present for the duration of the PI consultation group discussion, which took place over the course of 3 h. Three clinical researchers were present during the discussion (CJL, AML, VP), as described in Methods. From the analysis, three overarching themes were generated relating to i) the quality of the volunteer-facing written information, ii) how to improve the study design of both the uninfected sand fly biting study (FLYBITE) and the CHIM study (LEISH_Challenge), and iii) factors to motivate involvement in the research. The themes and their sub-themes, with supporting exemplar quotes, are provided in Additional File 1.

### Overarching theme 1: The quality of the volunteer-facing written information

This theme reflects on the participants’ perceived importance of the quality of the volunteer-facing material, in order to facilitate recruitment and engagement with the study. This overarching theme had three embedded themes: i) The need for clarity, ii) Consideration of the visual material used, iii) Consideration of written content.

#### The need for clarity

The participants described how further clarity was needed in the written volunteer-facing materials especially in relation to the extent of the health screening, length and number of study visits, and time involved versus remuneration. Clarity was also sought regarding inclusion and exclusion criteria, the comparative size of the parasite and sand fly, the size of possible scarring post-sand fly biting, anaphylaxis reaction in relation to the species of sand fly and its management, the burden of disease, and the number of sand flies and potential bites. One participant described a brief internet search of sand fly reactions as an example of the need for clarity:

*P7: Because I just Googled sand fly anaphylaxis and found Zane Mirfin writing about people, you know, I mean those aren’t the sand flies you’re using and it is completely anecdotal but if somebody’s looking at this and they could do exactly that, Google ‘sand fly’ and ‘anaphylaxis’ it does pull up anecdotes.*

*P9: You need to be clear that with this strain [of sand fly], with this one, these things are not, there is no recorded incidence of that [recurrence] happening. You need to be really clear.*

Additional areas requiring clarity related to the details of the FLYBITE study, including the quality of the sand fly and any possibility of transmissible infections from an ‘uninfected’ sand fly. PI consultation group participants also suggested for the eligibility requirements for LEISH_Challenge to be included in the initial FLYBITE study materials, in order to provide as much information for volunteers as possible. For the volunteer-facing information for the LEISH_Challenge study, the areas requiring clarity were the chances of contagion, infection and immunity, the treatment options available, and access to out-of-hours support. Suggestions regarding language choice and format of written information were also raised, as described by one participant:

*P7: And I think another thing with the literature was making it clear what the actual sort of, like the burden of the disease is. That it is this awful disfiguring disease you know and that’s what we want to stop. You’re not going to be getting that disease. What you’re doing is developing a model to study it …*

Further discussions around the specific terminology concerned the description of the biting process:

*P7: … saying you’re going to have a feeding chamber strapped to your arm just sounds a little bit sinister. *

*P2: I think feeding sounds a bit off-putting.*

*P7: Biting doesn’t sound so bad because they are going to be biting you.*

#### Consideration of the visual material used

This relates to images that may be provided of the sand fly, images of the cutaneous leishmaniasis lesion, and an example of the generalised scarring associated with an uninfected sand fly bite. These images were discussed as part of future advertisement and recruitment strategies, in order to provide clarity for potential volunteers, as described by one participant:

*P9: … it would be helpful if you put a photo to show of [the scar] afterwards. *

#### Consideration of the written content used

The inclusion of significant amounts of General Data Protection Regulation (GDPR) literature with information leaflets was raised. Participants voiced whether this could be condensed, and a link provided to more detailed GDPR resources (for example a weblink) in order to save time and reader fatigue. A discussion also took place regarding the inclusion of a plain English summary within the information leaflet itself and avoidance of scientific jargon. It was also suggested that the contribution to scientific knowledge should be emphasised within volunteer-facing material, as described by one participant:

*P9:… when you talk about the potential benefits of the study I think you need to add on that one of the potential benefits is you get to make a contribution, you get to make a difference in helping us create a model that will develop a vaccine that will change and possibly save lives. *

### Overarching theme 2) how to improve the study design (relating to FLYBITE and LEISH_Challenge studies)

A prolonged discussion of the various treatment options for cutaneous leishmaniasis took place between clinical investigators and PI consultation group participants. The discussion centred around the evaluation of preferred ‘bite site’ as well as existing treatment options including tablet, topical (typically creams and ointments), intra-lesional (that is, injected at the site of a cutaneous leishmaniasis lesion), intravenous therapies, cryotherapy, heat therapy and surgical excision. Treatment(s) would be necessary in the LEISH_Challenge study, but not the FLYBITE study.

This overarching theme had six embedded themes: (i) Alternative suggestions for volunteer engagement, (ii) ways to improve recruitment, (iii) suggestions for choice of bite location, (iv) choice of treatments (LEISH_Challenge study only), (v) informing the volunteers’ General Practioners (GPs) about their participation, and (vi) length of time in clinical environment.

#### Alternative suggestions for volunteer engagement

The participants suggested a number of alternative methods which could be considered for engaging volunteers to the study including grouped study inductions, volunteer experience videos, as well as websites and blogs. One participant shared their views on group-orientated sessions to be used in recruitment:

*P7: Yeah, I think group settings are actually very good for generating discussion and people will ask questions you haven’t thought of and you’ll ask questions they hadn’t thought of …*

*P7: … again, if you had a website you could have stories you know about people and the effect that it has on people’s lives.*

#### Improving recruitment

Ideas suggested included use of internet technologies such as email, weblinks and social media in combination with traditional ‘word-of-mouth’ and radio methods. Additional engagement that could reinforce recruitment could include interactive resources such as quizzes and automated pre-screening methods. Social media was favoured by one participant:

*P7: Facebook is one way that I know they’ve [other researchers] tried.*

#### Suggestions for location of sand fly biting and possible scarring site

The sand fly biting site suggested by the research team included the volar aspect of the forearm, close to the antecubital fossa (i.e. elbow pit). Participants then discussed their favoured areas, as well as enquiring about the relative propensity for scarring of the alternate areas. Several participants described a choice of anatomical areas as being an important factor in deciding to take part, but also the acceptability of any scarring:

*P7: It’s that thing about social acceptability is that lots and lots of people have got those vaccination scars on the tops of their arms and that’s just completely normal whereas a visible scar here it’s a bit, it’s just sort of human beings our acceptance of scars.*

*P5: I could make a suggestion you know our suggested site is here but if you like you can have it sort of elsewhere.*

*P1: So, you have a choice?*

*P5: Exactly, and ninety percent of the time they’ll just go with what you suggest because they don’t have a strong opinion they won’t care. But if they do sort of feel very strongly, great they get to actually feel in control.*

#### Choice of treatments

The treatment options are varied in the treatment of leishmaniasis, and participants were given the relative merits and shortcomings of each option. Participants agreed upon surgical excision as a possible method of treatment above other therapies. For example, several participants described their rationale for such a choice including as a reassuring treatment and for the scientific utility of having tissue that could be analysed by researchers. Surgical excision is described here by participants as a ‘biopsy’:

*P12: Would you think it was more reassuring to excise the lesion and use the ointment?...*

*… P9: Yes.*

*P1: So, if we kind of compromise and say from a study point of view our first choice is to take a biopsy so we can test it …*

*P3: I wouldn’t mind if I understood there was a benefit. If it says you’ll have a biopsy and I had a large birthmark and the reason I wouldn’t want excision is because having that off was quite unpleasant, but it was worth it for the biopsy results. I wouldn’t have had it off as a cosmetic procedure. So, I think if you’re advising people, we’re taking it off by excision because we’re going to do this to it rather than just for the sake of getting it*

*P7: So, a small biopsy is, if that’s part of the protocol. If you knew that was what you were signing up to. I mean I would be, personally I would be more than happy with that, you know, but that would be if it was what I had signed up to …*

#### Contacting the volunteer’s GP

Participants discussed the importance of confirming medical background history from the CHIM volunteer’s GP, and that it would actually be a reassuring practice, as described by one participant:

*P5: I think it’s good [contacting the volunteer’s GP] because for example with vaccine history a lot of people don’t necessarily know exactly what they had vaccines for whereas you know that will be in their medical records.*

#### Length of time in the clinical environment

Given the possible risk of reaction to sand fly bite, participants discussed that remaining in a clinical environment for continued observation post-sand fly biting would be reassuring, although did not favour an extended observation period:

*P9: … as long as the two hours would be reassuring to me that, you know, most anaphylactic reactions would happen within that time frame then that would make me reassured and happy.*

##### Overarching theme 3) motivations for involvement in research

Participants were asked about their motivations for involvement in the consultation exercise and if that translated into similar motivations for either the FLYBITE or the LEISH_Challenge studies. Three themes were generated within this overarching theme which included i) remuneration, ii) altruism/making a difference, iii) and dual motivation.

#### Remuneration

For many participants it was acknowledged that the financial aspect was an important draw for involvement in any clinical research. One participant described this as a boost to their income:

*P5: … it’s a very time effective way of supplementing their income. That’s why everyone I know took part in them [i.e. clinical trials], chose to take part in them.*

#### Altruism/making a difference

The renumeration for involvement was balanced by altruistic motivations for some participants. One of the participants described how altruism would be an important factor for potential volunteers

*P9: … I think there are a lot of people who want to get involved [in clinical trials] to make a difference as well*.

#### Dual motivation

Most participants voiced a mixture of motivations to be an important draw in taking part in either the FLYBITE study or LEISH_Challenge study, as discussed by some of the participants:

*P5: The altruism, it makes me feel better about taking part in it but it wouldn’t have been enough by itself.*

*P9: No, I think it’s very much both. I think a lot of people are motivated by both of those things it’s like I want to make a difference, I really want to make a difference but oh that’s great if I actually get some payment too that’s great. And I think one of the things when you talk about the potential benefits of the study I think you need to add on that one of the potential benefits is you get to make a contribution, you get to make a difference in helping us create a model that will develop a vaccine that will change and possibly save lives.*

## Discussion

Our adaptive approach to PI was effective in delivering the objectives of the consultation group and informed each of our proposed studies, FLYBITE and LEISH_Challenge. Overall, the proposed studies were well received by the PI consultation group, however this was only after clarifications around the language used were made, and on further debrief by clinical investigators. Participants held a frank discussion about the literature given in advance and made significant comments on the need to include our plain language summary within the body of any patient-facing material. Given the unique research question posed by CHIM studies, the complexity of neglected tropical diseases such as leishmaniasis and the possible implications for invasive intervention, this is a critical point within our research but also for clinical research as whole. Studies must be accessible to individuals, and often the impact of such interventions goes beyond those involved in studies themselves [30].

The concept of reflexivity in public engagement research is an important entity in examining inherent biases of researchers [31]. It is an ongoing process of evaluating the research experience and acknowledging that team members may individually analyse and interpret data based on their respective backgrounds and personal experience of the research topic. Given the complex nature of CHIM studies, reflexive practice is important in ensuring that participants with limited prior scientific knowledge were appropriately engaged and their opinions were allowed a platform. The clinical research team had no prior experience of CHIM studies, although have varied exposure to leishmaniasis studies and clinical research more broadly. The prior experience of clinical researchers ranged from some limited prior involvement, to significant input into clinical study design. The independent health psychologist chair and analyst were not involved directly in recruitment to the project but have had significant experience in practicing reflexivity. This heterogenous make-up of the team helped to therefore reduce research bias, and also reduce expectations of the themes to be generated. Given the unique mix of the research team, this was helpful in ensuring an open and encouraging environment where it was possible for research ideas to be openly questioned by participants.

The most important impact of the PI consultation group was the discussion of the treatment options posed. There is no clear consensus for a single treatment option for localised cutaneous leishmaniasis; it is mainly determined by species, local resources, experience of healthcare professionals and tolerability of treatment. After discussion of potential treatments, participants unanimously supported surgical excision of any cutaneous leishmaniasis lesion as the preferred treatment approach. Once a lesion is well established, excision is not currently favoured as a treatment option for localised cutaneous leishmaniasis amongst clinicians. However, early lesions are sometimes excised and tissue is examined as part of the diagnostic process. Excision as a treatment option in the context of the CHIM was therefore not discussed as a possibility prior to PI, in part due to expected poor acceptability. Participants also suggested that the cutaneous leishmaniasis lesion should be allowed to develop to the smallest possible size that could yield a positive identification of the cause whilst being distinguishable from incidental non-parasitic causes and also provide enough tissue material for analysis. These discussions concerning treatment options prompted further discourse with clinical specialists, experienced in the care of patients with leishmaniasis, who agreed with surgical excision as a valid method to terminate lesions in the context of the study. This has now been incorporated in the CHIM protocol, which has been approved following ethical review. This is therefore a significant change brought about by the public involvement process.

The research had some limitations. Although we did not record ethnicity as judged by participants themselves, it is an important issue more widely in research [32] and investigators noted a lack of ethnic diversity amongst our PI consultation group participants despite advertising widely. Leishmaniasis is present in tropical regions, typically low and middle incomes countries. It could be argued that the lack of representation on the PI panel may not be representative of a resource poor environment in an LMIC, although this PI consultation group was specifically concerned with studies to be conducted in the UK. Further public involvement work might be required if the CHIM study is adopted in LMICs. Skin colour can affect outcomes in several ways with respect to leishmaniasis, and indeed one study suggested that people of colour have increased morbidity due to disparities in access to healthcare [33]. Furthermore differing skin phototypes may have differing responses to a *Leishmania* infection, resulting in more scarring or other possible clinical sequelae such as hyper- or hypopigmentation [34]. These issues may alter health-seeking behaviour. A more ethnically diverse PI group may have had greater awareness of the importance of such issues. It was interesting however to note that one of the participants commented that they had ‘*mixed race children and my husband … he scars, you know’* [see Additional file 1]. Additionally, the majority of our participants were drawn from the local York area and were educated to university level, although this was not specifically recorded. York is considered one of the least deprived regions of the UK [35] and therefore the motivations for involvement in research may not reflect the general population. We were unable to recruit a participant who had had prior treatment for cutaneous leishmaniasis or had resolved their infection without treatment. However, the inclusion of such a patient may have added an extra layer of complexity to the discussion. The predominant cases of imported cutaneous leishmaniases to the UK are caused by New World *Leishmania* species that cause more protracted and aggressive lesions that are generally more difficult to treat than those caused by *Leishmania major,* the challenge agent for use in our project [14]. Participants who had undergone treatment for a species of *Leishmania* other than our proposed challenge agent would have had a different experience of treatment [36]. However, we were able to recruit participants of prior clinical research projects and a previous CHIM study participant.

Our research has some parallels with, and deviations from, other studies concerned with PI in general, and with respect to CHIM studies. It has been recognised for many years that public involvement is imperative to good research, however its implications and usage has been varied, dependent on setting, experience and local guidance and infrastructure [37, 38]. Furthermore, the literature suggests that where support for PI is strong, the implementation of outcomes within research is often weak [39]. This contrasts with our experience here, where our research project has been shaped in ways which would not have been possible without PI. A prominent example of PI within CHIMs have been discussed more recently with the advent of a CHIM for SARS-CoV-2, in order to test candidate vaccines [40, 41]. The themes generated within PI concerned with such studies, and points of discussion were broadly similar with our study; namely, risk to volunteers, level of remuneration, support networks and clarity of information [42]. The importance of CHIM studies being acceptable to the public, even after research ethics committee approval, has been discussed at length, and is an approach that we have mirrored here [43, 44]. This is demonstrated by our planned use of public involvement after our initial study, FLYBITE, and prior to our CHIM study, LEISH_Challenge, in a process of continual review. Further public involvement will take place at the end of the project in order to establish this CHIM as a standard model for use by researchers wishing to evaluate candidate leishmaniasis vaccines (see Fig. 1). Others have however importantly highlighted issues with particular respect to CHIM studies carried out in LMICs [43]. Some studies also discuss motivations for inclusion of volunteers in depth, characterizing the nature of altruism in CHIM studies and the need for balancing this with risk of the challenge agent [45]. One further important difference observed between our work here and others described in the literature is the discussion centred around treatment. Our PI activity involved participants in the conversation about the choice of treatment options to terminate the infection, something that has not been observed elsewhere as far as the authors can determine.

## Conclusion

We describe here the first detailed description of public involvement in controlled human infection research for cutaneous leishmaniasis. The PI consultation group session contributed not only to the project literature and highlighted public perception to study investigators, but also significantly impacted the design and practical considerations of the studies. Given the unique research question posed by controlled human infection studies, and the ethical debate surrounding them, the PI consultation group meaningfully improved understanding of these issues. We hope that this description will serve to improve awareness of the public involvement process and encourage researchers to utilise such processes to inform and improve study design and delivery.

## Supplementary Information

**Additional file 1.** Table of themes, with quotations.

**Additional file 2.** Guidance for Reporting Involvement of Patients and the Public (GRIPP2) reporting checklist.

## Data Availability

The datasets generated, analysed and supporting the conclusions of this study are available upon reasonable request from VP, CJL, AL GJ and PK.

## Abbreviations

GRIPP-2: Guidance for reporting involvement of patients and the public
GMP: Good manufacturing practices
LMIC: Low and middle-income country
NHS: National Health Service
NIHR: National Institute for Health Research
PI: Public involvement
