# Supplementary material for: Assessing public perception of a sand fly biting study on the pathway to a controlled human infection model for cutaneous leishmaniasis
Source: Res Involv Engagem. 2021 May 30;7:33. doi: 10.1186/s40900-021-00277-y (PMC8164890; doi:10.1186/s40900-021-00277-y)
Supplement: Supplementary file 1 — Additional file 1. Table of themes, with quotations. [file 40900_2021_277_MOESM1_ESM.docx]

**Additional File 1:** Table of themes, with exemplar quotations

| **Overarching Theme** | **Theme** | **Sub-theme 1** | **Sub-theme 2** | **Quotation** |
| --- | --- | --- | --- | --- |
| 1. Quality of participant-facing written information | 1.1 Need for clarity | 1.1.1 Anaphylaxis and management of this |  | *P7: Because I just Googled sand fly anaphylaxis and found Zane Mirfin writing about people, you know, I mean those aren’t the sand flies you’re using and it is completely anecdotal but if somebody’s looking at this and they could do exactly that, Google ‘sand fly’ and ‘anaphylaxis’ it does pull up anecdotes.*  *P7: But then there’s a risk of anaphylaxis with any vaccine which is given, which is why you stay in the doctor’s office and there are treatments available for anaphylaxis as well I mean how many deaths from anaphylaxis have there been from vaccination in the last year?*  *P9: So “There’s been no reported cases of anaphylactic reactions to sand fly bites in the whole of the medical literature, however there may still be a small risk of serious reaction to sand fly bite which may include breathing problems, widespread skin rash and rarely death.” So, it sounds to me reading that, I mean I know what anaphylactic is but if I didn’t, I would be reading that and going well there’s no reported cases but if it does happen, I’ll die. What you should be saying is there’s no reported cases so it’s extremely unlikely. If it does happen these are the symptoms. We can usually deal with them as well. You know you need to be really clear.*  *P5: Make it clear that there will be an observation period after any (intervention). There’s an observation period where you would see anaphylaxis if it was going to come up.*  *P9: I think as long as the two hours would be reassuring to me that, you know, most anaphylactic reactions would happen within that time frame then that would make me reassured and happy.* |

|  |  | 1.1.2 The nature of the parasite |  | *I don’t know whether it’s worth clarifying in the literature here what it is, what the parasite is because saying it’s a tiny parasite or a small parasite perhaps. (P7)*  *…Can the cutaneous one progress into visceral or are they too different? (P6?)* |
| --- | --- | --- | --- | --- |
|  |  | 1.1.3 The size of the scar |  | *P2: I think maybe the size of the scar would need clarifying because obviously if it’s a tiny scar you’re not going to notice on your arm. It’s quite different from having kind of a long running scar all the way down so I’m imagining a very very small scar.*  *P3: I was going to say if it’s less significant than a chickenpox scar, that’s the benchmark most of us have.* |
|  |  | 1.1.4 The species of sand fly |  | *You need to be clear that with this strain [of sand fly], with this one, these things are not, there is no recorded incidence of that [recurrence] happening. You need to be really clear. (P9)*  *Is there any chance that this disease could get spread by a different fly? (P7?)* |
|  |  | 1.1.5 The extent of health screening |  | *“P5: I think it’s worth mentioning in the information what it is that you’re going to be testing in the screening visit.*  *P7: And let them know that it can be seen as a potential benefit but there is also, because also you know with the vaccine trials, they do a pregnancy test for the women.”* |
|  |  | 1.1.6 Length and number of study visits |  | *…the actual information sheets you don’t list how long they’re going to be involved in it sort of clearly. There’s not a clear idea of how many visits. There’s not a clear idea of how much they’re going to get financially from it. So, from my perspective I wouldn’t be signing up to that because I don’t know. (P5)*  *P5: So, the length of time of the study doesn’t make a huge difference, it’s more down to the number of visits that are important.*  *P7: But it says here there will be four visits in total, each lasting between thirty minutes and two hours. No visit is going to last thirty minutes if the exposure is thirty minutes because you’ve got to arrive, and you’ve got to sign yourself in and you’ve got to get the gadget on and then. You’re not going to go in have it put on for thirty minutes and then go away.* |
|  |  | 1.1.7 Time involved versus remuneration |  | *the actual information sheets you don’t list how long they’re going to be involved in it sort of clearly. There’s not a clear idea of how many visits. There’s not a clear idea of how much they’re going to get financially from it. So, from my perspective I wouldn’t be signing up to that because I don’t know. (P5)* |
|  |  | 1.1.8 Inclusion and exclusion criteria |  | *A slight clarification for chronic skin conditions. You know I suffered from psoriasis as a teenager, I haven’t had psoriasis for years, but would that rule me out… (P7)*  *…do you kind of like make sure that your sample is like representative, do you try and get people of different genders and races and age groups… (P10)*  *A lot of researchers come to our group to talk about their projects and we ask them the same question [why is there an age limit of 65?] and usually the answer is well that’s just because it’s what most researchers do rather than it is a reason for this, it’s in the methodology or it’s to do with what the study. Because it is discriminatory if there isn’t a reason for it. (P9)* |
|  |  | 1.1.9 The human challenge model |  | *P7: And I think another thing with the literature was making it clear what the actual sort of, like the burden of the disease is. That it is this awful disfiguring disease you know and that’s what we want to stop. You’re not going to be getting that disease. What you’re doing is developing a model to study it…* |
|  |  | 1.1.10 The number of flies / lesions |  | *“P?: Maybe you could like clarify that in the patient information, just say there’s potential that you could get more than one lesion, just this is a consideration type thing.*  *P9: I think you need to add in how many sand flies will be put into the chamber, definitely.*  *P13: We can do that.*  *P?: And say that each fly will only bite once. Because that might be reassuring to people that there’s five.”* |
|  |  | 1.1.11 Specific to study 1 | 1.1.11.1 Quality of sand fly | *P4: Is it guaranteed that the uninfected flies, they definitely don’t carry any infection?* |
|  |  |  | 1.1.11.2 Eligibility for study 2 | *P11?: I think probably given that you’ve had an infection and possibly could have immunity we wouldn’t want then to put them into a study where they’ve got a new infection.*  *P5: Ok that’s something that’s worth sort of having in the information for them because you know if they’re sort of serial triallers they may go ok, this one I can get x amount for a couple of weeks or I could hold out for the next one and then do a full year trial and get a fair bit more.* |
|  |  | 1.1.12 Specific to study 2 | 1.1.12.1 Chances of contagion / infection / immunity | *…that’s really important. So, we need to be really clear it’s not contagious and that we can’t infect other people and you can’t move from one site to another. (P11)*  *“P1: One of the 4[…] potential features is once you’ve been infected and you’ve developed a lesion it may protect you in future against other types of leishmaniasis. Is that something, does anyone have any comments about that? Is it a very small point or-*  *P9: I think it’s worth saying. Because-*  *P1: It’s not clear cut.*  *P9: You may travel to a country that’s got it, great.*  *P13: We’re not sure about that.*  *[crosstalk]*  *P13: It’s possible, but I’m not too keen, I’m not sure we can prove that so I’d rather-*  *P?: But you don’t like it, ok.*  *P13: Too speculative.”* |
|  |  |  | 1.1.12.2 Treatment options | *…I think you need to just be clear that whatever treatment options you’re offering, they are all equally valid in terms of as a treatment. They’re all equally effective because that was something I was unsure of… (P9) (specific to study 2)*  *P9: whereas actually that if surgery could cut that bit out that seems better to me maybe …*  *P5: … once you’ve seen it, you know there is definitely a lesion there and you’re able to measure that, so three millimetres compared to five, why let it grow any larger?*  *P5: In terms of the length of time you’re allowing it to establish is there any reason to keep it for longer? For the purposes of the study is there any information that you can get out of that because from our perspective you know once you know it’s there, get it out of me. We’ve found out what we needed to know. Why bother leaving it?*  *P9: Which one of those would be best for lots of reasons, it’s not just about the scar.* |
|  |  |  | 1.1.12.3 Access to out-of-hours support | *If someone starts having health problems after they’ve left, say if it’s in the middle of the night, is there advice about who they should call say if it’s three in the morning or something? (P?)* |
|  |  | 1.1.13 Suggestions re: word choice | 1.1.13.1 Use of numbers rather than words | *…when you’re speaking, you’re using words like ‘tend to,’ do we have sort of like firm numbers, you know like in x numbers of experimental patients none had been seen to develop you know spreading lesions or whatever. (P7?)* |
|  |  |  | 1.1.13.2 Wording re: anaphylaxis | *…it was quite alarming reading there’s a very small risk of death. You see the word death and it kind of puts you off. (P?)*  *…use the term there is observation and treatment available to mitigate the risk… (P5)* |
|  |  |  | 1.1.13.3 Description of ‘feeding’ chamber | *…saying you’re going to have a feeding chamber strapped to your arm just sounds a little bit sinister. (P7?)*  *“P2?: I think feeding sounds a bit off-putting.*  *P7?: Biting doesn’t sound so bad because they are going to be biting you.”* |
|  |  |  | 1.1.13.4 Description of species | *P9: I was just going to say one of the first things that struck me when I read that was that you need to say that, because when you’re calling it the what did you call it the Leishmania major that sounds really, this doesn’t sound benign or mild at all and that wasn’t clear when I read it. I had to read into the fact that in the context of what you were saying it must be a weaker strain sort of quite benign but that’s not actually made clear. I think people would just read that word.* |
|  |  |  | 1.1.13.5 Wording re: skin reaction | *“P5: Do you think perhaps you could just give us an example of what you mean by significant reaction perhaps.*  ***I: Graphically? Pictures?***  *P5: You could, or just sort of state you know.*  *P12: Redness, swelling.*  *P5: Exactly, yeah.”* |
|  |  | 1.1.14 Suggestions re: format of written information | 1.1.14.1 Use of procedure flow chart | *…so, if you say total time and then have the actual flow chart of what you’re going to be doing… (P?)* |
|  |  |  | 1.1.14.2 Too much white space | *…there’s a huge amount of white space of these sheets, you know formatting wise. You know it doesn’t need to be like that. (P7)* |
|  |  |  | 1.1.14.3 Use of bullet points to improve clarity | *…having those bullet points that volunteers must actually be like that [i.e. inclusion criteria] is much clearer than having it in paragraph form and reading those things as a paragraph. I think it’s actually much clearer to bullet point that. (P9)* |
|  | 1.2 Consideration of visual material used | 1.2.1 Image of sand fly important vs unimportant |  | *You could include it if you wanted but I don’t think it’s going to make a lot of difference. (P5)*  *…seeing a picture of the sand fly too. I want to know what’s biting me. (P9)* |
|  |  | 1.2.2 Image of lesion to correspond with text (study 2) |  | *…I’d reflect that [the size of the lesion] in the text because otherwise you’ve read three to five millimetres and you think ooh that’s really quite small and then you see it [the image] and it’s almost twice the size that you’ve been mentally prepared for. (P?)* |
|  |  | 1.2.3 Imp. to show bites |  | *…the little picture of bites again, it’s reassuring… (P?)* |
|  |  | 1.2.4 Imp. to show image of scarring (study 1) |  | *…it would be helpful if you put a photo to show of [the scar] afterwards. (P9)* |
|  |  | 1.2.5 Imp. to include image of feeding chamber |  | *This is a really new concept to people that isn’t in their everyday experience, so I think to talk about it and then illustrate it is really helpful because they’ll see oh that’s what is actually going to happen, that’s what it looks like. (P9)* |
|  |  | 1.2.6 Suggest editing image of researcher |  | *…it might be worth cropping the photo [of the researcher’s coincidental scar on her arm]. (P?)* |
|  | 1.3 Consideration of written content used | 1.3.1 Data protection information | 1.3.1.1 Could be more concise / balanced | *“P9: It makes the patient information sheet really long getting through all the stuff about data protection. I think you could summarise it together in one section, but you chose to spread out more, it could be a bit more concise because it just makes the sheet very hard to read.*  *P7: And it also makes it look like your safety isn’t as important as your data.*  *P9: I think once it got to the rodent-protected boxes I was a bit kind of I’m not sure all of that needed to go in.”* |
|  |  |  | 1.3.1.2 Suggested link to website | *“P?: Or a link to a website.*  *P12?: Yeah, I was about to say that do you think we could make it much more succinct and link it to a website.*  *P?: Yes. And then we could get rid of all.”* |
|  |  | 1.3.2 Transferring info. from ‘plain English summary’ |  | *…lots of points were unclear in there [the PIS] but they were much clearer in the plain English summary. There’s lots of stuff you could just add on and tweak this to make this a lot clearer. (P9)*  *P7: I absolutely agree, I think the details from the plain English summary need to be there.*  *P9: And the fact that you created a plain English summary which wasn’t the thing you were then telling the patients is of concern to me as a first thing. Because it’s like why would that be more complicated? Because the simple information that that’s got in it should be the plain English summary.* |
|  |  | 1.3.3 Emphasise contribution to scientific knowledge |  | *…when you talk about the potential benefits of the study I think you need to add on that one of the potential benefits is you get to make a contribution, you get to make a difference in helping us create a model that will develop a vaccine that will change and possibly save lives. (P9)* |
| 2. How to improve study design | 2.1 Alternative suggestions for patient engagement | 2.1.1 Group discussion (study induction) |  | *P1: Do you think it’s more useful to have it in a group setting with volunteers rather than one on one?*  *P7: Yeah, I think group settings are actually very good for generating discussion and people will ask questions you haven’t thought of and you’ll ask questions they hadn’t thought of …* |
|  |  | 2.1.2 Patient experience video (study induction) |  | *P5: …a little one-off video just a little one-minute video explaining.*  *P7?: I mean that must be worth so much reassurance to most people looking through it you know there she is with the thing strapped onto her arm.*  *P9: Absolutely. I agree completely.*  *P9: Yeah I take your point that hopefully as you develop these materials they become much clearer and much more informative and people feel much clearer about it all but I think what you said earlier and I think maybe for the first visit the people that are taking part in the study watch a video.* |
|  |  | 2.1.3 Patient experience blog |  | *…one thing which was effective was a blog by somebody who’d taken part in a vaccine trial. If the person who’s been involved, you know the person who’s got those bites, the young lady in is it Israel or the Czech Republic? If she, if you have a website where you could be directed to for example her describing what it was like because that means a lot to certain people, not everyone obviously, but a lot of people if you can read somebody’s narrative of doing it, it makes it a lot more… (P7)* |
|  |  | 2.1.4 Study website |  | *…again, if you had a website you could have stories you know about people and the effect that it has on people’s lives. (P7)* |
|  | 2.2 Ways to improve recruitment | 2.2.1 Email / link sharing |  | *P7: …I get sent a lot of adverts to look at.*  *P5: Exactly, that’s what’s my primary way of getting into it now.* |
|  |  | 2.2.2 Facebook |  | *P7: Facebook is one way that I know they’ve tried.* |
|  |  | 2.2.3 Local radio |  | *P5: They advertised on the radio, local radio quite a bit as well.*  *P7: Yeah local radio.* |
|  |  | 2.2.4 Word of mouth |  | *P5: It does seem to be word of mouth mostly in the end. You know once you’ve got one or two people into it, they tend to bring more.* |
|  |  | 2.2.5 Other methods |  | *…back in Oxford there’s a mailing list that you can sign up to in the fresher’s fair for everyone who’s interested in vaccine trials and so on, so you get updated every term on which ones are available… (P5)*  *…possibly having a stall in the fresher's fair so you can get incoming students to sign up for something like that just as something in future. (P5)* |
|  |  | 2.2.6 Use of pre-trial quiz |  | *P7: …this [the use of a quiz] would be really good, especially if you’re doing an online recruitment because if people do an online you know application you can then just steer them straight into a quiz.*  *P5: And then it also filters it out quite nicely before you actually have the job of meeting people in person.*  *P7?: Yeah that that person has actually read and understood the literature rather than they’re just somebody who’s applying.”* |
|  |  | 2.2.7 Use of automated pre-screen |  | *“P5: …the automated [pre-screen], it does work very well, I’m just thinking it’s a good way once they’re already interested.*  *P7: Once they’re committed, yeah.”* |
|  | 2.3 Suggestions for choice of bite location / scarring |  |  | *P7: Yes, because keloid scarring was something that I was going to ask about because yeah because I’ve got mixed race children and my husband he scars, you know.*  *P7: It’s that thing about social acceptability is that lots and lots of people have got those vaccination scars on the tops of their arms and that’s just completely normal whereas a visible scar here it’s a bit, it’s just sort of human beings our acceptance of scars.*  *P9: I think part of it’s the psychology is that yourself, you will see that and you will see that bit of you but actually ironically other people would see that bit of you more than I think they look at that. I wouldn’t notice you’d got that; I might notice more if you’d got a scar there.*  *“P5: I could make a suggestion you know our suggested site is here but if you like you can have it sort of elsewhere.*  *P?: So you have a choice?*  *P5: Exactly, and ninety percent of the time they’ll just go with what you suggest because they don’t have a strong opinion they won’t care. But if they do sort of feel very strongly, great they get to actually feel in control.”* |
|  | 2.4 Choice of treatments (study 2) |  |  | *…are any of the treatments different in what they’re actually doing to the infection because I think that’s significant too. (P9)*  *“P12: Would you think it was more reassuring to excise the lesion and use the ointment?*  *P9: Yes.”*  *“P12: …I’m really interested that our colleague from Oxford said “get it out” so does that mean you feel it would be best to cut it out?*  *P?: That’s my instinct.*  *P3: Excision wouldn’t be my first choice.”*  *P1: So, if we kind of compromise and say from a study point of view our first choice is to take a biopsy so we can test it…*  *P3: I wouldn’t mind if I understood there was a benefit. If it says you’ll have a biopsy and I had a large birthmark and the reason I wouldn’t want excision is because having that off was quite unpleasant, but it was worth it for the biopsy results. I wouldn’t have had it off as a cosmetic procedure. So, I think if you’re advising people, we’re taking it off by excision because we’re going to do this to it rather than just for the sake of getting it*  *P7: So, a small biopsy is, if that’s part of the protocol. If you knew that was what you were signing up to. I mean I would be, personally I would be more than happy with that, you know, but that would be if it was what I had signed up to…* |
|  | 2.5 Contacting GP |  |  | *I think it’s good [contacting the volunteer’s GP] because for example with vaccine history a lot of people don’t necessarily know exactly what they had vaccines for whereas you know that will be in their medical records. (P5)*  *I think most people want to know if you’re contacting their GP. (P9)* |
|  | 2.6 Length of time in clinical environment |  |  | *…as long as the two hours would be reassuring to me that, you know, most anaphylactic reactions would happen within that time frame then that would make me reassured and happy. (P9)* |
| 1. Motivations for involvement in the research | 3.1 Remuneration |  |  | *…it’s a very time effective way of supplementing their income. That’s why everyone I know took part in them [i.e. clinical trials], chose to take part in them. (P5)* |
|  | 3.2 Altruism / making a difference |  |  | *…I think there are a lot of people who want to get involved [in clinical trials] to make a difference as well. (P9)* |
|  | 3.3 Dual motivation (altruism plus remuneration) |  |  | *“P5: The altruism, it makes me feel better about taking part in it, but it wouldn’t have been enough by itself.*  *P9: No, I think it’s very much both. I think a lot of people are motivated by both of those things it’s like I want to make a difference, I really want to make a difference but oh that’s great if I actually get some payment too that’s great. And I think one of the things when you talk about the potential benefits of the study I think you need to add on that one of the potential benefits is you get to make a contribution, you get to make a difference in helping us create a model that will develop a vaccine that will change and possibly save lives.* |
